# Authenticated Timing Protocol Based on Galileo ACAS

**DOI:** 10.3390/s22166298

**Published:** 2022-08-21

**Authors:** Francesco Ardizzon, Laura Crosara, Nicola Laurenti, Stefano Tomasin, Nicola Montini

**Affiliations:** 1 Department of Information Engineering, University of Padova, Via Gradenigo 6/A, 35131 Padova, Italy; 2 Qascom, Via Marinali 87, 36061 Bassano del Grappa, Italy

**Keywords:** GNSS, CAS, OSNMA, timing, security

## Abstract

Global navigation satellite systems (GNSSs) provide accurate positioning and timing services in a large gamut of sectors, including financial institutions, Industry 4.0, and Internet of things (IoT). Any industrial system involving multiple devices interacting and/or coordinating their functionalities needs accurate, dependable, and trustworthy time synchronization, which can be obtained by using authenticated GNSS signals. However, GNSS vulnerabilities to time-spoofing attacks may cause security issues for their applications. Galileo is currently developing new services aimed at providing increased security and robustness against attacks, such as the open service navigation message authentication (OS-NMA) and commercial authentication service (CAS). In this paper, we propose a robust and secure timing protocol that is independent of external time sources, and solely relies on assisted commercial authentication service (ACAS) and OS-NMA features. We analyze the performance of the proposed timing protocol and discuss its security level in relation to malicious attacks. Lastly, experimental tests were conducted to validate the proposed protocol.

## 1. Introduction

Timing and synchronization are two key services provided by global navigation satellite systems (GNSSs). GNSS receivers use ranging signals and satellite-reported time information to obtain a position, velocity, and time (PVT) solution, providing time with nanosecond-level accuracy [1]. Moreover, synchronization between receivers at different locations can be established and maintained using GNSS reference time, such as coordinated universal time (UTC) or the Galileo system time (GST). Thanks to this level of accuracy, several sectors rely on GNSS for synchronization operations, from financial institutions that use GNSS to timestamp transactions to Industry 4.0 and Internet of things (IoT) applications. The main standards for the dissemination of time and frequency over digital networks are the network time protocol (NTP) and the precision time protocol (PTP). The accuracy of NTP is usually within tens of milliseconds over the Internet, and it can be less than 1 ms in local area networks (LANs) with ideal network conditions [2], while PTP provides better accuracy, from hundreds of nanoseconds to microseconds [3]. Consequently, when nanosecond-level accuracy is required, a good solution is to adopt a local time server that relies on GNSSs.

For many of the mentioned applications and others that will be considered, accurate, reliable, and trustworthy time synchronization is required, and it can be obtained by relying only on authenticated GNSS signals. Indeed, the authentication feature provides trustfulness as it incorporates specific features that cannot be predicted or falsified into the broadcast GNSS signals, and an authentication-enabled receiver can interpret these characteristics to distinguish authentic signals from forgeries. The authentication can take place at two complementary levels: at the data level, i.e., on navigation messages, and at the ranging level, on pseudoranges between the satellite and receiver. The combination of data and code authentication enables the calculation of a secure PVT solution.

Navigation message authentication (NMA) techniques aim to ensure the authenticity of the content of the navigation messages, providing the user with the integrity protection of data. Open service navigation message authentication (OS-NMA) is a data authentication function for public Galileo E1B signals [4] in which the message transmitted by the satellites is interleaved with authentication data generated through broadcast authentication protocol timed-efficient stream loss-tolerant authentication (TESLA) [5], suitably adapted for optimal transmission via Galileo [1,6]. The TESLA protocol employs a one-way chain shared by Galileo satellites with a public root key. The keys in the chain are used in reverse order to generate message authentication codes (MACs). Keys are then shared (always in reverse order) in broadcast mode with a delay of a few seconds. The receiver can verify the MACs as soon as it becomes aware of the key.

Securing the pseudorange measurements computed by the receiver means authenticating the signal’s source and the time that it takes for the signal to reach the receiver. Spreading code encryption (SCE) techniques are the most reliable option to limit access to GNSS signals, as they render the spreading code unpredictable. Some SCE-type solutions in the literature are the P(Y) code for GPS and the commercial authentication service (CAS) for Galileo, which complement OS-NMA by offering spreading code level authentication in the E6 band. The assisted commercial authentication service (ACAS), recently presented in [7,8], provides a code authentication method that is based on navigation data received and authenticated by OS-NMA, including the key to generate the digital signature. This is part of Galileo commercial service (CS). A change in the SCE approach for public GNSS signals was proposed in [9], where a spreading code authentication (SCA) technique was proposed that authenticates a transmitted signal by watermarking the public spreading code with unpredictable sequences. A similar SCA technique was proposed in [10], where short sequences called spread spectrum security codes (SSSCs) were interleaved with the public spreading code. This approach was refined in [11,12], where the authentication scheme called chips-message robust authentication (CHIMERA) was introduced, which aims at jointly authenticating the navigation data and the spreading code of GPS signals for civil usage. This scheme replaces a small part of the spreading code with a secret, cryptographically generated sequence that can subsequently be reproduced by the receivers when they become aware of the key. In this context, a way to optimize trade-offs between security level and signal availability to receivers that do not know the modified code was derived in [13]. In the following, we focus on the combination of OS-NMA and ACAS.

In this paper, we introduce a secure timing protocol that relies solely on E6C authentication features and OS-NMA authenticated messages. We used E6C ACAS to build a clock model that is both robust and thus able to compute reliable time corrections, and secure since it could detect signal tampering. Our approach comprised two consecutive steps: first, the receiver processes the E6C measurements to estimate the receiver clock bias and drift; second, the receiver combines the obtained measurements to estimate the current clock bias by either using a Kalman filter, or fitting a linear or quadratic least squares model. Moreover, we propose strategies for timing attack detection in which we check the consistency of each new measurement with the model that had been calculated. We look at two approaches for this task: clock monitoring and innovation testing. We model a time-push attack to validate the performance of the proposed security checks. Moreover, we evaluate the proposed protocol on both simulated and experimental data collected with a professional GNSS receiver in nominal conditions and under-attack scenarios.

The rest of the paper is organized as follows. Section 2 briefly reviews the main concepts of the ACAS mode; then, the scenario for our analysis is described in Section 3. The main contribution of this paper is provided in Section 4, where we describe our proposed approach for secure ACAS-based timing, while the attack and its detection are described is Section 5. Simulation and experimental results are discussed in Section 6. Lastly, Section 7 draws the conclusions of the paper.

## 2. Review of ACAS

CAS is the Galileo’s SCE service aiming at providing signal authentication without modifications to Galileo first-generation core infrastructure and signals, and requiring only minimal changes to both the system and the receiver. CAS is currently under development but expected to be established by 2024: in particular, a proposal known as ACAS was presented in [7,8]. In ACAS, the E6C pseudo-random noise (PRN) spreading codes are neither short nor periodic sequences, but are generated by the system as a stream known as encrypted code sequence (ECS). Part of the ECS is re-encrypted using the TESLA keys employed by the OS-NMA protocol, and disseminated with the E1 open signal, generating the re-encrypted code sequence (RECS). The RECS are stored and published at predefined times on servers accessible to the public, such as the GNSS service center (GSC). Together with RECS, the server also publishes additional useful files for PVT computation, such as the broadcast group delay (BGD) for the E1–E6 bands. Once the RECS are retrieved from the server, the user can decrypt them by using the corresponding TESLA key, obtaining the related ECS. Lastly, the ECS is tested against previously stored samples received from the E6C signal, allowing for the user to verify the authenticity of the received signals. The TESLA key related to one (or more) RECS is revealed within the public Galileo E1B signal with a few seconds of delay compared to the transmission of the latter by the satellites.

This approach enables the receiving user to operate in standalone mode for the validity period of the predownloaded data (i.e., the RECS files) and without storing any secret cryptographic key. The RECS lengths are defined by the number of chips in these sequences, which is one of the key parameters in ACAS design as it determines the duration of the signal fragment used in correlation during the acquisition phase. Together with the size of the bins used for the Doppler frequency search, they define the acquisition search space and thereby the ability to find correlation peaks from which the pseudoranges and the authenticated PVT solution are computed. Another key parameter in ACAS is the distance between two consecutive RECS sequences, which determines how often the receiver can compute an authenticated solution. However, with ACAS, users assess the authenticity of the signals by checking the consistency between E6 and E1, which is not authenticated at the ranging level. In this work, we propose an authenticated timing protocol that relies only on ACAS and the navigation messages, which are both authenticated.

The PVT solution calculated via ACAS may also be useful for initializing the time synchronization required by OS-NMA, as RECSs files are designed to include the transmission time associated with the corresponding ECS of the keystream E6C, which can be used to resynchronize the receiver. The default ACAS operating mode is snapshot mode, since no navigation message and thus no ephemeris data are transmitted on E6.

## 3. System Model

**Figure 1:**
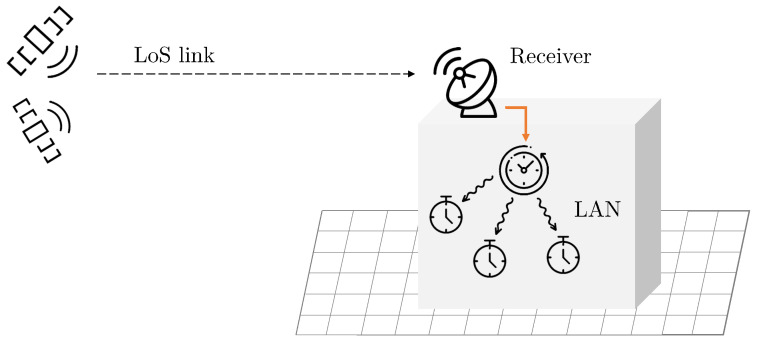
Pictorial representation of the considered scenario.

We consider a scenario where a master clock is responsible for the synchronization of a network, composed of several devices or sensors connected via LAN. We assumed that this network was isolated; therefore, no attacker could influence the time dissemination process. The master clock is connected to a GNSS receiver, for instance, by being placed on the roof of a building with clear view of the sky. For this reason, we may assume that the received signals are transmitted by satellites mostly in line-of-sight (LOS), and that the effects of the multipath are minimal. The antenna position was fixed and known. We examined the case of a single-antenna receiver. Multiple antennas may still be employed, for example, to enhance the performance or security of the scheme by, e.g., checking the angle of arrival of a GNSS signal [14,15]. A representation of the considered scenario is depicted in Figure 1.

**Figure 2:**
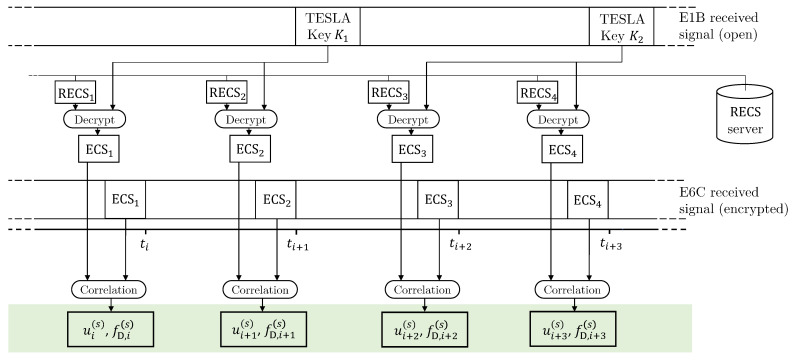
Summary of ACAS operations at the receiver side for signal transmitted by satellite *s*.

We considered a multifrequency receiver enabled to acquire and track Galileo signals (at least) in bands E1 ($f_{c}=1575.42$ MHz) and E6 ($f_{c}=1278.75$ MHz). Moreover, the receiver exploits both Galileo OS-NMA and ACAS. As briefly described in Section 2 and depicted in Figure 2, once the RECS files are published in the server and the TESLA key is received, the receiver decrypts the RECSs by using the corresponding key to obtain a local replica of the ECSs. Next, for the subset of Galileo satellites in view S⊆{1,2,…24}, it correlates the local replica with the prerecorded Galileo E6C signal samples and, from the correlation peaks, it computes code delay $u_{i}^{\left(s_{i}\right)}$ and the Doppler frequency $f_{D,i}^{\left(s_{i}\right)}$, measured by the receiver on signal on band E6, transmitted by satellite $s_{i}\in S$ and received at time $t_{i}$.

After collecting *M* observables, the aim is to estimate the current master clock bias. In detail, at time $t_{0}$, we used *M* measurements collected from the satellites in S at times $t_{1},\ldots ,t_{M}$, with $t_{i+1}\geq t_{i}$ and $t_{0}>t_{M}$. Unlike the PVT computation, the proposed protocol provides timing even with fewer than four satellites in view.

## 4. Proposed Approach

**Figure 3:**
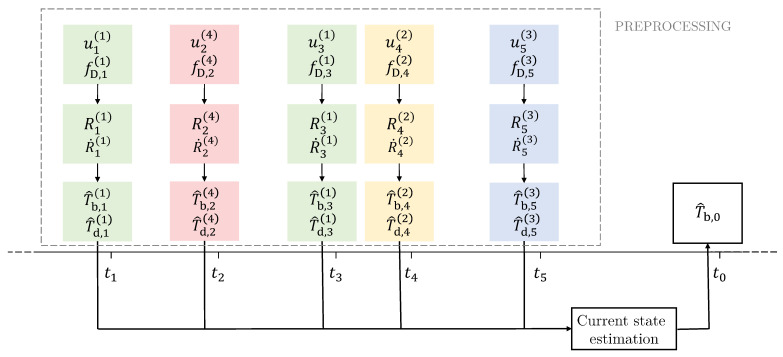
Schematic representation of preprocessing and current-state estimation phases.

In this section we propose a protocol that relies only on the observables authenticated by ACAS and the message, authenticated instead by OS-NMA, to compute the master clock correction. An ACAS observation is a 4-ple $O_{i}=\left\{t_{i},s_{i},u_{i},f_{D,i}\right\}$, where $t_{i}$ is the observation time, $s_{i}\in S$ is the satellite ID, $u_{i}$ is the observed code delay, and $f_{D,i}$ is the observed frequency offset (Doppler shift). We define the set of observables O as

(1) $$O=\left\{O_{i}:\;i=1,\ldots ,M\right\}=\left\{\left(t_{i},s_{i},u_{i},f_{D,i}\right):\;i=1,\ldots ,M\right\},$$

with |O|=M, where all the measurements are obtained from the E6 signal. In the *preprocessing phase*, from observation in O, we derived ${\hat{T}}_{b,i}$, estimated the clock bias at time $t_{i}$ on the basis of observation $O_{i}$, and ${\hat{T}}_{d,i}$, and estimated the clock drift at time $t_{i}$ on the basis of observation $O_{i}$. So, the output of the preprocessing phase is the set

(2) $$T=\{\left({\hat{T}}_{b,i},{\hat{T}}_{d,i}\right):\;i=1,\ldots ,M\},$$

which had the same cardinality as O. Each measurement in T, indexed by i=1,…,M, may be acquired by a different satellite. Next, the *current-state estimation* phase follows where measurements in T are used to compute the master clock correction, at time $t_{0}$, ${\hat{T}}_{b,0}$. Figure 3 summarizes both the two phases.

The last phase concerns *security checks*, where we tried to detect anomalous estimates ${\hat{T}}_{b,0}$ of the clock bias; we considered the *clock monitoring* and *innovation test* as solutions for this task.

### 4.1. Preprocessing

Starting from each code delay measurement $u_{i}\in O$, following the procedure described in [16], we computed pseudorange $R_{i}$ at time $t_{i}$ associated with satellite $s_{i}$. As indicated in Section 3, receiver position $P_{\mathrm{rx}}\left(t\right)$ is fixed and publicly known. Satellite position $P_{\mathrm{sat}}\left(t\right)$ and clock bias $T_{b,\mathrm{sat}}\left(t\right)$ can be retrieved from the authenticated OS-NMA message; thus, both can be considered to be authenticated at any time *t*. The computed pseudorange can be decomposed as

(3) $$R_{i}=r_{i}+c\left(T_{b,i}-T_{b,\mathrm{sat}}^{\left(s_{i}\right)}\left(t_{i}\right)\right)+D_{i}+\eta_{i},$$

where $r_{i}=\left|\right|P_{\mathrm{sat}}^{\left(s_{i}\right)}\left(t_{i}\right)-P_{\mathrm{rx}}\left(t_{i}\right)\left|\right|$ is the geometric range, $T_{b,\mathrm{sat}}^{\left(s_{i}\right)}\left(t_{i}\right)$ is the satellite $s_{i}$ clock bias, $D_{i}=D_{\mathrm{iono}}^{\left(s_{i}\right)}\left(t_{i}\right)+D_{\mathrm{tropo}}^{\left(s_{i}\right)}\left(t_{i}\right)$ is the sum of ionospheric and the tropospheric delays, $\eta_{i}$ is the additional noise due to the signal processing errors and multipath, and *c* is the speed of light.

The pseudoranges computed for E1 are corrected by using the estimations of ionosphere and troposphere delays transmitted in the E1B navigation message. In this case, we worked with E6 pseudoranges: however, since the troposphere is a nondispersive medium, the corrections for the tropospheric delay of band E1, ${\hat{D}}_{\mathrm{tropo},E1}^{\left(s\right)}\left(t\right)$, and E6, ${\hat{D}}_{\mathrm{tropo},E6}^{\left(s\right)}\left(t\right)$ were identical for all s∈S. On the other hand, if the ionosphere is instead a dispersive medium, given the correction for E1, the correction for E6 is [17]

(4) $${\hat{D}}_{\mathrm{iono},E6}^{\left(s\right)}\left(t\right)={\hat{D}}_{\mathrm{iono},E1}^{\left(s\right)}\left(t\right)\frac{f_{E1}^{2}}{f_{E6}^{2}},$$

for all s∈S and for every time instant *t*. Correction ${\hat{D}}_{\mathrm{iono},E1}^{\left(s\right)}\left(t\right)$ must be obtained through a proper ionospheric correction model such as the Klobuchar model [18], or more precise models, such as Galileo NeQuick [19] or the IRI-P 2017 [20]. Only the measurements from E6 were actually authenticated; therefore, we could not exploit the measurements from another band (e.g., E1 or E5) to remove the ionospheric delay contribution, as it is typically performed in multifrequency GNSS receivers; instead, we had to use the model computed by using the parameters in the authenticated navigation message.

The receiver clock bias estimation at time $t_{i}$ is then calculated from (3) and (4) as

(5) $${\hat{T}}_{b,i}≜\frac{1}{c}\left(R_{i}-r_{i}-{\hat{D}}_{i}\right)+T_{b,\mathrm{sat}}^{\left(s_{i}\right)}\left(t_{i}\right)=T_{b,i}+\xi_{b,i},$$

where $T_{b,i}$ is the real receiver clock bias at time $t_{i}$, and $\xi_{b,i}$ is the clock bias estimation error taking into account the error residuals due to the nonperfect atmospheric delays estimation and the additional noise component $\eta_{i}$.

Next, we compute the *pseudorange rate* ${\dot{R}}_{i}$ at time $t_{i}$ as

(6) $${\dot{R}}_{i}=-\lambda f_{D,i},$$

where $f_{D,i}$ belongs to the authenticated observables set O and λ is the wavelength of E6. From (3), the pseudorange rate can then be decomposed as

(7) $${\dot{R}}_{i}={\dot{r}}_{i}+c\left(T_{d,i}-T_{d,\mathrm{sat}}^{\left(s_{i}\right)}\left(t_{i}\right)\right)+\gamma_{i}+{\dot{\eta }}_{i},$$

where

(8) $$\gamma_{i}=\gamma^{\left(s_{i}\right)}\left(t_{i}\right)\;,\;\gamma^{\left(s\right)}\left(t\right)≜\frac{\partial }{\partial t}\left[D_{\mathrm{iono}}^{\left(s\right)}\left(t\right)+D_{\mathrm{tropo}}^{\left(s\right)}\left(t\right)\right]$$

is a term modeling both the time derivatives of the the atmospheric delays and the signal processing errors. Moreover, the geometric range derivative ${\dot{r}}^{\left(s\right)}\left(t\right)$ is given by

(9) $$\begin{array}{c} {\dot{r}}^{\left(s\right)}\left(t\right)=\frac{\partial }{\partial t}\left|\right|P_{\mathrm{sat}}^{\left(s\right)}\left(t\right)-P_{\mathrm{rx}}\left(t\right)\left|\right|={\left(v_{\mathrm{sat}}^{\left(s\right)}\left(t\right)-v_{\mathrm{rx}}\left(t\right)\right)}^{T}\frac{P_{\mathrm{sat}}^{\left(s\right)}\left(t\right)-P_{\mathrm{rx}}\left(t\right)}{\left|\right|P_{\mathrm{sat}}^{\left(s\right)}\left(t\right)-P_{\mathrm{rx}}\left(t\right)\left|\right|}\\ ={\left(v_{\mathrm{sat}}^{\left(s\right)}\left(t\right)-v_{\mathrm{rx}}\left(t\right)\right)}^{T}e^{\left(s\right)}\left(t\right)=v_{\mathrm{LOS}}^{\left(s\right)}\left(t\right), \end{array}$$

where $e^{\left(s\right)}\left(t\right)$ is the unit vector that points to the receiver antenna from the satellite, so $v_{\mathrm{LOS}}^{\left(s\right)}\left(t\right)$ is the velocity projected into the LOS direction. Moreover, $v_{\mathrm{rx}}\left(t\right)=0\;\forall t$, since the position of the GNSS receiver is fixed. Thus, term ${\dot{r}}_{i}$ appearing in (7) is obtained as

(10) $${\dot{r}}_{i}={\dot{r}}^{\left(s_{i}\right)}\left(t_{i}\right)=v_{\mathrm{LOS}}^{\left(s_{i}\right)}\left(t_{i}\right).$$

Analogously to (5), we compute

(11) $${\hat{T}}_{d,i}≜\frac{1}{c}\left({\dot{R}}_{i}-v_{\mathrm{LOS}}^{\left(s_{i}\right)}\left(t_{i}\right)\right)+T_{d,\mathrm{sat}}^{\left(s_{i}\right)}\left(t_{i}\right)=T_{d,i}+\xi_{d,i},$$

where $T_{d,i}$ is the real receiver clock drift at time $t_{i}$ and $\xi_{d,i}$ is the clock drift estimation error. Repeating this procedure for i=1,…,M, we obtain the set T.

It is possible to statistically model both $\xi_{b,i}$ and $\xi_{d,i}$. A partial model for the first term is provided in [7,21,22]; however, the second-order descriptions of $\xi_{b,i}$ and $\xi_{d,i}$ are sufficient for the analysis in this paper.

### 4.2. Current-State Estimation

In the previous section, we showed how to derive measurements in T starting from the authenticated observables in O. These estimates are exploited to compute the actual receiver clock bias that is used to correct the master clock. The design of a specific algorithm for this task is justified, since the clock bias and drift estimations are relative to time $t_{i},i=1,\ldots ,M$; therefore, we need a model that exploits the past measurements to compute the current one. Moreover, past measurements are affected by noise, modeled by $\xi_{b,i}$ and $\xi_{d,i}$. We analyzed three different approaches to this task: a least squares (LS) quadratic model, a LS linear model, and a Kalman filter.

#### 4.2.1. LS-Quadratic and Linear Model

The first two solutions leverage the idea that clock bias increases (or decreases) over time following a parabola, where the quadratic term, with coefficient *drift rate*, is expected to have a low impact. For instance, considering the *time of ephemeris* $t_{\mathrm{oe}}$, the Galileo satellite clock bias is computed as follows [23]

(12) $$T_{b,\mathrm{sat}}^{\left(s\right)}\left(t\right)=a_{0}^{\left(s\right)}+a_{1}^{\left(s\right)}\left(t-t_{\mathrm{oe}}\right)+a_{2}^{\left(s\right)}{\left(t-t_{\mathrm{oe}}\right)}^{2},$$

where $a_{0}^{\left(s\right)}$, $a_{1}^{\left(s\right)}$, and $a_{2}^{\left(s\right)}$ represent the satellite clock bias, clock drift, and clock drift rate measured at time $t_{\mathrm{oe}}$, respectively. Typically the drift rate is transmitted to as $a_{2}^{\left(s\right)}=0$, leading to a de facto linear model. Thus, we consider both a quadratic and a linear model.

Analogously to (12), calling $\tau_{i}=t_{0}-t_{i}$ the time difference between the current time at which we want to compute the clock bias estimation and the time associated to the measurements, we can write

(13) $$\begin{array}{cc} {\hat{T}}_{b,i}= & a_{0}+a_{1}\tau_{i}+a_{2}\tau_{i}^{2}+\epsilon_{b,i}, \end{array}$$

(14) $$\begin{array}{cc} {\hat{T}}_{d,i}= & a_{1}+2a_{2}\tau_{i}+\epsilon_{d,i}, \end{array}$$

where $a_{0}$, $a_{1}$ and $a_{2}$ are now the parameters modeling the receiver clock behavior, ${\hat{T}}_{b,i}$ and ${\hat{T}}_{d,i}$ are the measurements in T computed in the preprocessing phase, $\epsilon_{b,i}$ and $\epsilon_{d,i}$ are the estimation errors related to the *i*-th measurement. Equivalently to (13) and (14), in matrix form, we have

(15) $$\left(\begin{array}{c} {\hat{T}}_{b,i}\\ {\hat{T}}_{d,i} \end{array}\right)=\left(\begin{array}{ccc} 1 & \tau_{i} & \tau_{i}^{2}\\ 0 & 1 & 2\tau_{i} \end{array}\right)\left(\begin{array}{c} a_{0}\\ a_{1}\\ a_{2} \end{array}\right)+\left(\begin{array}{c} \epsilon_{b,i}\\ \epsilon_{d,i} \end{array}\right)=\left(\begin{array}{c} E_{b,i}\\ E_{d,i} \end{array}\right)a+\epsilon_{i},$$

where $a={\left[a_{0}\;a_{1}\;a_{2}\right]}^{T}$ is the vector of parameters we aim to estimate. Next, considering all the measurements in T, we stack the matrices, obtaining

(16) $$y=\left(\begin{array}{c} y_{b}\\ y_{d} \end{array}\right)=\left(\begin{array}{c} E_{b}\\ E_{d} \end{array}\right)a+\left(\begin{array}{c} \epsilon_{b}\\ \epsilon_{d} \end{array}\right)=Ea+\epsilon ,$$

where $y_{b}$ and $y_{d}$ are the columns vectors collecting the *M* bias and drift measurements, respectively, in T, $E_{b}={\left[E_{b,1}^{T},\ldots ,E_{b,M}^{T}\right]}^{T}$ and $E_{d}={\left[E_{d,1}^{T},\ldots ,E_{d,M}^{T}\right]}^{T}$ contain the time difference terms associated to each measurement in $y_{b}$ and $y_{d}$, respectively, and $\epsilon ={\left[\epsilon_{1},\ldots ,\epsilon_{M}\right]}^{T}$. In order to minimize the mean square error (MSE), we performed the estimation by using the pseudoinverse

(17) $$\hat{a}={\left(E^{T}E\right)}^{-1}E^{T}y,$$

and we obtained the estimations of clock bias and drift at time $t_{0}$ as

(18) $$\begin{array}{cc} {\hat{T}}_{b,0} & ={\hat{a}}_{0}, \end{array}$$

(19) $$\begin{array}{cc} {\hat{T}}_{d,0} & ={\hat{a}}_{1}. \end{array}$$

An analogous derivation can be performed starting from a linear model, replacing (15) with

(20) $$\left(\begin{array}{c} {\hat{T}}_{b,i}\\ {\hat{T}}_{d,i} \end{array}\right)=\left(\begin{array}{cc} 1 & \tau_{i}\\ 0 & 1 \end{array}\right)\left(\begin{array}{c} a_{0}\\ a_{1} \end{array}\right)+\left(\begin{array}{c} \epsilon_{b,i}\\ \epsilon_{d,i} \end{array}\right).$$

#### 4.2.2. Kalman Filter

In this section, we investigate the use of a Kalman filter to estimate the bias. In particular, every time a new estimate $\left\{{\hat{T}}_{b,i},{\hat{T}}_{d,i}\right\}$ was available, we updated the model and perform a new prediction; moreover, even when no new measurement was available, we exploited the previously trained model to estimate the current clock correction. A more detailed description of the Kalman filter can be found in [24].

The procedure was divided into two phases, *prediction* and *model update*. We call $x_{i}$ the *true state* at time $t_{i}$, and $z_{i}$ the *input* at time $t_{i}$, that is,

(21) $$x_{i}=\left(\begin{array}{c} T_{b,i}\\ T_{d,i}\\ {\dot{T}}_{d,i} \end{array}\right),\;z_{i}=\left(\begin{array}{c} {\hat{T}}_{b,i}\\ {\hat{T}}_{d,i} \end{array}\right),$$

where ${\dot{T}}_{d,i}$ represents the clock drift rate, which we did not measure directly. Then, the *state-transition matrix* and the *observation matrix* are given by

(22) $$F_{i}=\left(\begin{array}{ccc} 1 & t_{i}-t_{i-1} & {\left(t_{i}-t_{i-1}\right)}^{2}\\ 0 & 1 & 2(t_{i}-t_{i-1})\\ 0 & 0 & 1 \end{array}\right),\;H_{i}=\left(\begin{array}{ccc} 1 & 0 & 0\\ 0 & 1 & 0 \end{array}\right).$$

Differently from the general model for the Kalman filter, we had no control input. In the prediction step, we computed a priori state estimate ${\hat{x}}_{i|i-1}$ and its covariance matrix $P_{i|i-1}$:

(23) $${\hat{x}}_{i|i-1}=F_{i}{\hat{x}}_{i-1|i-1}$$

(24) $$P_{i|i-1}=F_{i}P_{i-1|i-1}F_{i}^{T}.$$

Calling $R_{i}$ the measurement noise covariance, during the update step, we computed

(25) $$y_{i}=z_{i}-H_{i}{\hat{x}}_{i|i-1}$$

(26) $$B_{i}=H_{i}P_{i|i-1}^{-1}H_{i}^{T}+R_{i}$$

(27) $$K_{i}=P_{i|i-1}H_{i}^{T}B_{i}^{-1}$$

(28) $${\hat{x}}_{i|i}={\hat{x}}_{i|i-1}+K_{i}y_{i}$$

(29) $$P_{i|i}=\left(I_{2}-K_{i}H_{i}\right)P_{i|i-1}.$$

We call ${\hat{x}}_{i|i}$ and its covariance $P_{i|i}$ the updated a posteriori estimate of the state. Term $y_{i}$ is called *innovation* and is used together with its covariance $B_{i}$ during the innovation check in the security steps. Repeating this procedure for every measure in T, we obtained the *M*-th estimation $x_{M}$. Then, from (23), we computed the a posteriori estimation at time $t_{0}$ as ${\hat{x}}_{0|M}=F_{0}{\hat{x}}_{M|M}$, where

(30) $$F_{0}=\left(\begin{array}{ccc} 1 & t_{0}-t_{M} & {\left(t_{0}-t_{M}\right)}^{2}\\ 0 & 1 & 2(t_{0}-t_{M})\\ 0 & 0 & 1 \end{array}\right).$$

Lastly, ${\hat{T}}_{b}$ is the first element of ${\hat{x}}_{0|M}$.

## 5. Timing Attack and Detection

In the system model of Section 3, we assumed that the position of the GNSS receiver was fixed and publicly known. Therefore, the receiver was assumed to perform a consistency check on the received signal, such that, if the receiver PVT computation yielded a position much different from the expected one or a significant velocity, an alarm would be raised. Moreover, since the satellites’ position was known, the receiver could reject any signal coming from satellites that should not be in view: thus, the attacker is also forced to generate signals corresponding only to satellites actually in view by the legitimate receiver. Hence, the attacker knows that (1) all the attacks causing a relevant change in the victim’s computed position or velocity are detected, and (2) signals transmitted by satellites that should not be in view by a legitimate receiver are neglected.

For these reasons, we consider an attacker performing a *time-push* attack: this is a *meaconing* attack where the receiver records signals and retransmits them with additional delays, adding an equal bias in all pseudoranges, which results in error in the time calculation of the PVT solution by the receiver, while the computed position does not change, as is proven in Section 6. Moreover, this attack may indeed target ACAS, where the signal cannot be tracked since the receiver operates in snapshot mode: this grants the attacker a time window to record the signal and perform a time-push attack. Sudden changes in the estimated clock bias may alert the receiver: thus, the attacker performs a time push in a smoothly progressive manner, gradually increasing the delay. However, to be effective, the attacker must be close to the victim’s antenna to have the same satellites in view of the legitimate receiver.

A possible countermeasure to prevent this attack would be to render the area around the receiver inaccessible by, for instance, installing surveillance cameras and/or surrounding the building with a fence. Still, we considered a worst-case scenario where the attacker managed to approach close enough to the receiver antenna and isolate the legitimate receiver, ensuring that only fake signals are received to perform the time-push attack.

To detect the presence of false measurements among the obtained corrections, we considered *clock-monitoring* and *innovation-testing* [25,26] methods. Formally, we frame this problem as hypothesis testing: considering null-hypothesis $H_{0}$ as the nominal condition where the signals are transmitted by the legitimate transmitter, the receiver observes a test statistic, β, and decides whether β is compatible with $H_{0}$ or not.

### 5.1. Clock Monitoring

As discussed in Section 4.2, the receiver clock bias is typically assumed to have either linear or quadratic behavior over time: we can then analyze the clock bias corrections over time and if anomalous discontinuities are detected we raise an alarm. This is the idea behind clock-monitoring techniques. Given the clock model ${\hat{a}}^{\prime }$ estimated through either (15) or (20) at time $t_{i}-\delta$, i.e., the previous epoch, it is possible to compute a prediction $\left\{{\tilde{T}}_{b,i},{\tilde{T}}_{d,i}\right\}$ of the measurements at time $t_{i}$, as

(31) $$\left(\begin{array}{c} {\tilde{T}}_{b,i}\\ {\tilde{T}}_{d,i} \end{array}\right)=\left(\begin{array}{ccc} 1 & \delta & \delta^{2}\\ 0 & 1 & 2\delta \end{array}\right){\hat{a}}^{\prime }.$$

Hence, for bias and drift, we adopted as the test statistic the quantities

(32) $$\beta_{b,i}≜{\tilde{T}}_{b,i}-{\hat{T}}_{b,i},$$

(33) $$\beta_{d,i}≜{\tilde{T}}_{d,i}-{\hat{T}}_{d,i},$$

and test

(34) $${\hat{H}}_{i}=\left\{\begin{array}{c} H_{0}\;\mathrm{if}\;\left|\beta_{b,i}\right|<\lambda_{b}\mathrm{and}\left|\beta_{d,i}\right|<\lambda_{d},\\ H_{1}\;\mathrm{otherwise}, \end{array}\right..$$

where thresholds $\lambda_{b}$ and $\lambda_{d}$ are chosen a priori by the user as a predefined false alarm (FA) probability. When a specific attack model is available, it may be possible to instead set the thresholds on the missed detection (MD) probability. More in detail, considering, for instance, drift threshold $\lambda_{d}$, it may be worth taking into account the actual clock specifications, thus evaluating a bound of the clock drift in nominal conditions [27].

If the distribution of the tests statistics $\beta_{b,i}$ and $\beta_{d,i}$ were known, it would be possible to replace (34) with two generalized likelihood ratio tests (GLRTs); however, the statistical characterization of such quantities is out of the scope of this work and is left to future works. Lastly, while we show the effectiveness of the clock monitoring only in relation to the LS models, such techniques may also be employed with the Kalman filter.

### 5.2. Innovation Testing

While using the Kalman filter, during the update step, each prediction is corrected by innovation term (25) that, in steady-state conditions, has mean and covariance

(35) $$E[y_{i}]=0$$

(36) $$\mathrm{COV}(y_{i})=B_{i}.$$

We can then use the normalized innovation as a test statistic, computed as follows:

(37) $$\beta_{K,i}=y_{i}^{T}B_{i}y_{i}.$$

In nominal conditions, $\beta_{K,i}$ is assumed to have chi-squared distribution [26] with as many degrees of freedom as the size of the measurement $z_{i}$, $\beta_{K,i}∼\chi^{2}$. Thus, to assess the authenticity of the measurement, we could use the GLRT test against a uniform distribution

(38) $${\hat{H}}_{i}=\left\{\begin{array}{c} H_{0}\;\mathrm{if}\;p\left(\beta_{K,i}|H_{0}\right)\geq \lambda_{k}\\ H_{1}\;\mathrm{otherwise} \end{array}\right.,$$

where $\lambda_{k}$ is chosen by the user to match a predefined FA probability.

## 6. Results and Discussion

In this section, first, we validate the proposed approach; next, we show that the time-push attack described in Section 5 is successful even if a legitimate receiver knows its actual position, highlighting the need for additional security checks.

We collected experimental data to build the set of authenticated observables O serving as input for the preprocessing phase. The detection capabilities of the methods proposed in Section 5.1 and Section 5.2 were tested against a simulated time-push attack.

### 6.1. Validation Using Experimental Data

**Figure 4:**
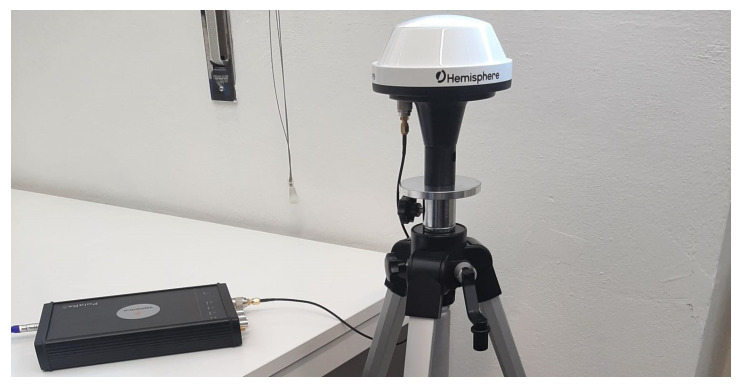
Setup used for the experimental dataset collection: Septentrio PolarRx5 receiver connected to an A42 Hemisphere antenna.

To validate the proposed approach described in Section 4 we performed experimental tests collecting signals from an open-sky environment with a Septentrio PolarRx5 receiver connected to a A42 Hemisphere antenna. The experimental setup is depicted in Figure 4.

The output of the receiver was logged using the Septentrio binary format (SBF) standard and postprocessed after the experiments, obtaining a dataset of measurements from different constellations and frequency bands, summarized in Table 1.

We considered only measurements from two Galileo satellites that were visible during the whole experiment. As *ground truth* $T_{b}$ that was later used to evaluate the goodness of our estimates ${\hat{T}}_{b}$, we used the clock bias measurements calculated from the PVT solution computed by the receiver using the whole set of measurements available in the dataset: on average, the PVT was computed by the receiver using the signal coming from 16 satellites. The Septentrio PolaRx5 is equipped with a voltage-controlled and temperature-controlled crystal oscillator (VCTCXO). Since only E6C ranging measurements were authenticated, we set the receiver to use the Klobuchar ionospheric correction model, which is the one typically used for GNSS receivers, estimating the ionospheric delay as in (4). More precise sophisticated models as Galileo NeQuick [19] and IRI-P 2017 [20] can be employed. For the sake of simplicity, we show that even the simpler Klobuchar model is enough to obtain satisfactory results, showing our method’s robustness. Next, we extracted set O from our dataset considering only the measurements from E6C.

**Figure 5:**
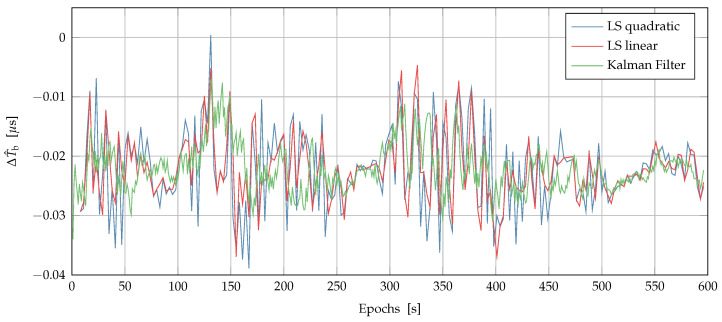
Difference between the ground truth and the clock estimations, $\hat{\Delta }T_{b}$, obtained by using the LS quadratic, LS linear and the Kalman filter on the experimental data.

Figure 5 shows the master clock bias estimation error as the difference between the ground truth and the clock estimations, $\Delta {\hat{T}}_{b}$, obtained using the LS quadratic, LS linear estimation methods and the Kalman filter in Figure 5. The LS methods described in Section 4.2.1 were used to compute one clock bias estimation ${\hat{T}}_{b}$ every 2 s using the 4 most recent available measurements, so that M=4. The Kalman filter computed one new estimate ${\hat{T}}_{b}$ every second. All the tested methods were effective, achieving an error limited to less than 50 ns, obtaining precise timing with fewer than four satellites in view.

### 6.2. Numerical Results and Attack Detection

To simulate the attacks, we used our signal generator and software receiver developed for the MORE Galileo open service signal integrity protection (MORE GOSSIP) project, funded by the European Space Agency (ESA) (see also [28]). We simulated the Galileo E6 baseband signal (the carrier frequency still influenced the Doppler frequency), generating both data (E6B) and pilot (E6C) components as in Galileo specifications [23], modulated with a BPSK(5), i.e., with code frequency $f_{\mathrm{code}}=5.115$ MHz. We considered an additional linear (deterministic) clock drift of 0.5 parts per million (ppm). We modeled a noiseless scenario with RECS duration equal to the PRN code length on E6, i.e., 5115 chips. Concerning CAS, we assumed that one new RECS would be disclosed every second. We generated 5 channels, i.e., 5 signals from five different satellites with 16 bit quantization. The sampling frequency was set to $f_{s}=2f_{\mathrm{code}}=10.23$ MHz, and each simulation scenario lasted for 100 s. On the receiver side, the acquisition was performed by using the same sampling frequency, and the Doppler bin size was set to 75 Hz. The receiver collected measurements $\left\{{\hat{T}}_{b,i},{\hat{T}}_{d,i}\right\}$ with a frequency of 1 Hz; as indicated before, since we assumed that the one RECS was made public every 60 s, we used only one of the measurements of the satellite in view per acquisition round as input for the model.

#### 6.2.1. Nominal Scenario

We start by considering legitimate dataset $H_{0}$. Only one RECS is disclosed at every epoch; thus, only one signal every epoch can be used to update the state.

**Figure 6:**
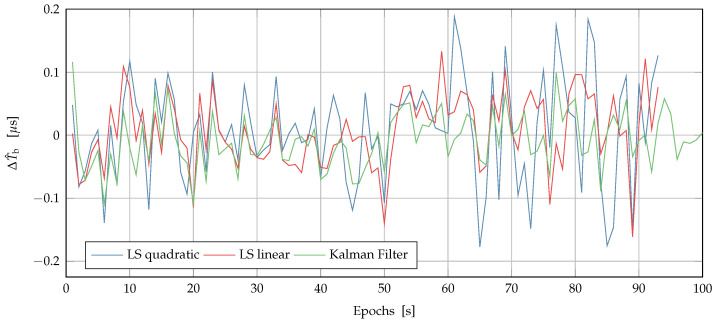
Difference between the ground truth and the clock estimations, $\Delta {\hat{T}}_{b}$, obtained by using the LS quadratic, LS linear and the Kalman filter on the simulated data.

Figure 6 shows the results obtained for the current-state estimation phase described in Section 4.2. In particular, we show $\Delta {\hat{T}}_{b}$, i.e., the difference between ground truth and clock estimations obtained by using the LS quadratic, LS linear, and the Kalman filter: all the methods were effective, achieving maximal deviation lower than 200 ns and a zero mean even using only one (new) measurement per epoch (i.e., per minute). Thus, all the methods could be employed for this task.

#### 6.2.2. Attack Scenario

In this section, we evaluate under-attack scenarios, such as the ones described in Section 5.

In the first part of this section, we show the impact of a time-push attack, proving that such attacks cannot be detected just by the check on the receiver position. In the second part, we discuss the performance of the clock-monitoring and innovation-check methods, showing the different behaviors of the test statistics $\beta_{b}$, $\beta_{d}$, and $\beta_{K}$ in the legitimate and under-attack scenarios, i.e., $H_{0}$ and $H_{1}$.

As indicated in Section 5, a sudden spike in the estimated clock bias may alert the receiver; thus, the attacker introduces the delays in a ramplike fashion. We modeled a scenario where the attacker managed to isolate the victim receiver and acquired only the forged E6 signals.

**Figure 7:**
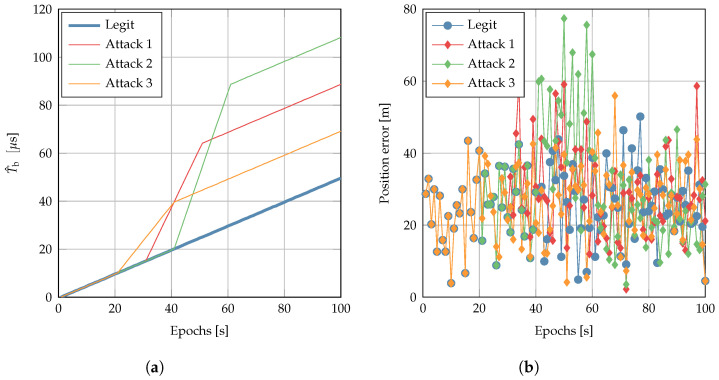
Comparison of legitimate and under-attack scenarios for (**a**) clock bias and (**b**) positioning error obtained using the simulated dataset.

Figure 7 reports the results: while the positioning error statistic was indeed indistinguishable in $H_{0}$ and $H_{1}$, the impact on the clock bias is clear. This confirms that we cannot trust the timing obtained on a PVT that passes by the naive position check. Hence, we suggest dedicated algorithm and strategies specifically designed for secure timing.

Next, we validate the security checks described in Section 5 considering a legitimate scenario and three attack scenarios. Each attack lasted 20 s with a constant drift of 1, 2 and 3 ppm, and achieved a final delay of 20, 40, and 60 μs, respectively. Each attack started at a different time.

**Figure 8:**
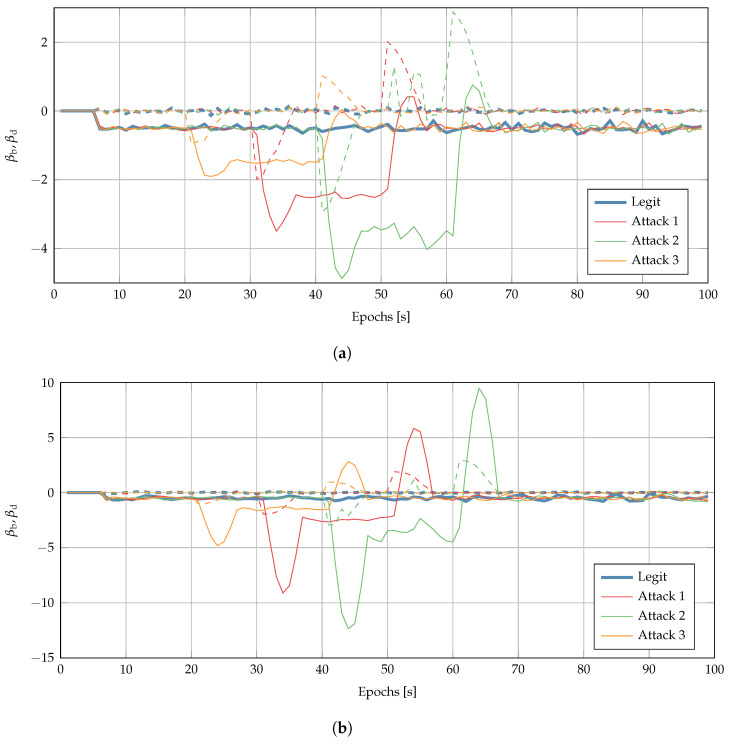
Test statistics, $\beta_{b}$ (continuous lines), and $\beta_{d}$ (dashed lines) used by clock monitoring: comparison of legitimate (thick blue) and under-attack scenarios for the (**a**) linear and (**b**) quadratic LS models.

Figure 8 shows the test statistic obtained via clock monitoring in nominal conditions and an under-attack scenario: both $\beta_{b}$ and $\beta_{d}$ presented spikes associated to the start and end of the attack, which had a magnitude much greater than the standard deviation of the same test statistic in the nominal conditions. This test was, thus, indeed effective in detecting time-push attacks, since it is easy for the user to set a threshold to distinguish legitimate from under-attack scenarios. Moreover, performing more tests, it could be possible for the user to fine-tune the threshold by observing the receiver operating characteristic (ROC) curves.

**Figure 9:**
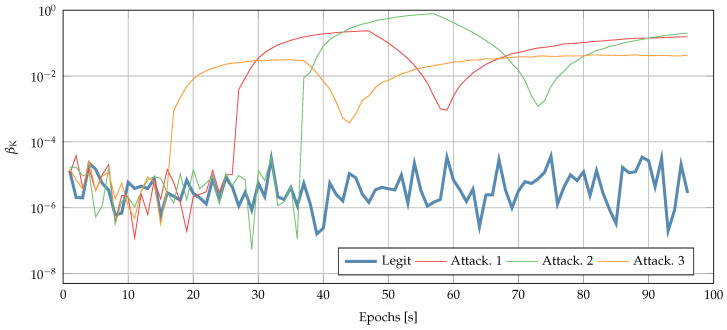
Test statistic $\beta_{K}$ used by the innovation testing: comparison between legitimate (blue) and under-attack scenarios.

Figure 9 shows the test statistic $\beta_{K}$ used for the innovation testing and described in Section 5.2. A jump is presented when the attacker starts (and ends) the time-push attack. Therefore, this technique is also successful at detecting time-push attacks.

## 7. Conclusions

In this work, we presented a secure timing protocol that may be used, for instance, by Industry 4.0 applications to synchronize multiple IoT devices within a facility. We considered a scenario where the master clock was securely connected to a GNSS receiver, and all the devices or sensors aimed to be synchronized. The protocol was based upon the new Galileo ACAS protocol and relied only on authenticated measurements to obtain the clock correction.

The procedure was composed by three blocks: first, exploiting the fact that the facility position is known, the receiver processes the E6C measurements to obtain an estimation of the receiver clock bias and drift; second, the receiver merges the previously obtained measurements to compute the current clock bias estimation by fitting either a linear or a quadratic least-squares model, or by using a Kalman filter. Lastly, we also considered the employment of a security evaluation phase where we tested the consistency of each new measurement with the previously estimated model. For this task, we considered two methods: clock monitoring and innovation test. We validated the proposed procedure using an experimental dataset collected with a Septentrio PolaRx5 receiver, and simulated data considering both legitimate and under-attack conditions. The obtained numerical and experimental results show that our protocol was both able to compute a reliable timing correction and to reject time-push attacks.

## Figures and Tables

**Table 1:** Constellations and central frequencies of the measurements collected in the experimental dataset.

	Central Frequency, $f_{c}$ [MHz]								
	1176.45	1207.14	1227.60	1245.5	1278.75	1268.52	1561.098	1575.42	1601.5
**Galileo**	E5a	E5b			E6			E1BC	
			L2 C/A					L1 C/A	
**GPS**	L5		L2 P(Y)					L1 P(Y)	
**Beidou**	B2a	B2l				B3l	B1l	B1C	
**GLONASS**				L2 C/A					L1 C/A

## Data Availability

Not applicable.

## Abbreviations

ACAS	Assisted commercial authentication service
BGD	Broadcast group delay
CAS	Commercial authentication service
CHIMERA	Chips-message robust authentication
CS	Commercial service
ECS	Encrypted code sequence
FA	False alarm
GLRT	Generalized likelihood ratio test
GNSS	Global navigation satellite system
GSC	GNSS service center
GST	Galileo system time
IoT	Internet of Things
LAN	Local area network
LOS	Line of sight
LS	Least squares
MAC	Message authentication code
MD	Missed detection
MSE	Mean square error
NMA	Navigation message authentication
NTP	Network time protocol
OS-NMA	Open service navigation message authentication
ppm	Parts per million
PRN	Pseudo-random noise
PTP	Precision time protocol
PVT	Position, velocity, and time
RECS	Re-encrypted code sequence
ROC	Receiver operating characteristic
SBF	Septentrio binary format
SCA	Spreading code authentication
SCE	Spreading code encryption
SSSCs	Spread spectrum security codes
TESLA	Timed-efficient stream loss-tolerant authentication
UTC	Coordinated universal time
VCTCXO	Voltage-controlled and temperature-controlled crystal oscillator
